# Recruitment challenges in physiotherapy trials

**DOI:** 10.1186/1745-6215-16-S2-P95

**Published:** 2015-11-16

**Authors:** Varsha Gandhi, Cushla Cooper, Karen Barker

**Affiliations:** 1 University of Oxford, Oxford, UK; 2 Nuffield Orthopaedic Centre, Oxford University Hospitals NHS Trust, Oxford, UK

## 

Challenges and barriers to recruitment in surgical and drug trials have been well documented. However, in exercise programme-based studies, there is scarce literature on the challenges to recruitment. Here we highlight recruitment barriers unique to large, randomised-controlled, multi-centre physiotherapy trials through the ‘PROVE’ trial; Physiotherapy rehabilitation for Osteoporotic Vertebral Fracture.

PROVE is the largest ongoing trial in outpatient rehabilitation in the UK with a target of 600 participants. The aim is to assess the effect of physiotherapy treatments on the quality of life of patients who suffer back pain due to osteoporotic vertebral fractures. Patients are randomised to one of the three treatment groups, viz. exercise therapy, hands-on manual therapy and usual care (control group). The patients participate for 12 months during which there are three assessments.

Problems at site setup included acknowledgement of the validity of the research hypothesis by physiotherapy managers, acceptance of the proposed treatment interventions and changing current set treatment strategies. Even after site setup there were significant challenges in establishing inter-disciplinary links within the hospital for participant identification, defining research roles within the study and poor maintenance of screening logs. Participant barriers included frequency of treatment visits and difficulty in hospital accessibility.

We illustrate the rehabilitation specific recruitment barriers in outpatient physiotherapy multi-centred RCT along with successful strategies implemented, resulting in participation on course to meet the target recruitment (currently at 250 participants across 20 sites).

